# Comparison of Total Hip Arthroplasty after Two Types of Failed Hip Preserving Procedures with Primary Total Hip Arthroplasty

**DOI:** 10.1111/os.12618

**Published:** 2020-01-20

**Authors:** Wei Zuo, Jin‐hui Ma, Wei Cui, Wan‐shou Guo, Wei Sun

**Affiliations:** ^1^ Peking University China‐Japan Friendship School of Clinical Medicine Beijing China; ^2^ Center for Osteonecrosis and Joint Preserving & Reconstruction, Department of Orthopaedic Surgery China‐Japan Friendship Hospital Beijing China

**Keywords:** Total hip arthroplasty, Porous tantalum implant (PTI), Bone impaction grafting (BIG), Hip preserving procedures

## Abstract

**Objective:**

Porous tantalum implantation (PTI) and bone impaction grafting (BIG) through a window at the femoral head neck junction are known as two types of joint‐preserving procedures. They provide an alternative option in the treatment of osteonecrosis of the femoral head by providing strong structural support to the subchondral plate. However, when earlier joint‐preserving treatments fail, conversion to a total hip arthroplasty seems to be the final treatment of choice. This emphasizes the importance of joint‐preserving procedures that do not hinder the clinical results of a subsequent total hip arthroplasty. The results of conversion total hip arthroplasty after failed PTI and BIG are still controversial. The purpose of this study was to compare the clinical and radiological outcomes of total hip arthroplasty after failed PTI or BIG surgery with primary total hip arthroplasty.

**Methods:**

Patients at our institution between 2010 and 2014 who underwent total hip arthroplasty after failed PTI or BIG surgery compared to primary total hip arthroplasty were retrospectively reviewed. A total of 27 patients (30 hips) who underwent total hip arthroplasty after failed PTI surgery (group I) were matched according to age, gender and BMI index with 28 patients (30 hips) who underwent total hip arthroplasty after failed BIG surgery (group II) and 30 patients (30 hips) who underwent primary total hip arthroplasty (group III). The clinical results included preoperative and postoperative Harris Hip score, surgery duration, blood loss volume and clinical complications. Radiological follow‐up results included abduction angle and anteversion angle of the acetabular cup, periprosthetic osteolysis, and prosthesis subsidence.

**Results:**

There was no significant difference in the preoperative and postoperative Harris Hip score among the three groups at the latest follow‐up (*P* = 0.247). The surgery duration was longer and intra‐operative blood loss volume was higher in group I than in group II and group III (*P* < 0.05, respectively). There was no difference in surgery duration and blood loss volume between group II and group III (*P* > 0.05). There was no significant difference in radiological follow‐up results among the three groups (*P* > 0.05). Varying degrees of residual tantalum debris were seen on postoperative radiographs of all group I patients. There was no difference in the incidence of complications among the three groups (*P* > 0.05).

**Conclusions:**

PTI group had higher blood loss volume and surgery duration than BIG group and primary total hip arthroplasty group. BIG group had no significant differences with primary total hip arthroplasty group in clinical and radiological follow‐up results. There were no significant differences between the three groups in the Harris Hip score and radiological follow‐up results.

## Introduction

Osteonecrosis of the femoral head is a common refractory and progressive disease usually affecting young and middle‐aged orthopaedic patients. If effective treatment strategies are not used, it could eventually lead to femoral head collapse and degenerative changes to the hip joint. Hip joint preserving surgery has attracted more and more attention due to the fact that the long‐term clinical results of total hip arthroplasty are still unsatisfactory in young and middle‐aged patients and tend to have complications such as prosthesis dislocation and loosening^1^. Therefore, the rate of hip joint revision is high with a risk of complications. Hip joint preserving surgery should slow down or even prevent the progress of femoral head collapse and degenerative changes. The aim is to postpone total hip arthroplasty as long as possible.

There are a large number of operative procedures in clinical practice for preserving hip joints including core decompression, transtrochanteric rotational osteotomy, vascularized fibula grafting, porous tantalum implant (PTI), and bone impaction grafting (BIG). BIG and core decompression, which have simple operative procedures, can relieve the internal pressure of the femoral head and pain symptoms, but they cannot remove necrotic bone completely. Therefore, they are used for the early stage of osteonecrosis of the femoral head. The application of vascularized fibula grafting fills the defects of the former. It can not only remove the necrotic bone completely, but also provide the graft bone with nourishing blood vessels for the femoral head, which is conducive to the reconstruction of the bone structure in the femoral head. The application of tantalum rod provides mechanical support for the femoral head and has the function of preventing the collapse of the femoral head. Transtrochanteric rotational prevents the secondary collapse of the femoral head by rotated out of the weight‐bearing area of the acetabulum.

The treatment of osteonecrosis of the femoral head should be individualized^2^, ^3^. The collapse of the femoral head is an important distinguishing factor. Many studies indicated that hip preserving procedures cannot achieve the expected clinical results for patients with collapsed femoral head^4^, ^5^, ^6^. Hence, total hip arthroplasty is the first choice. Our early clinical results showed that individualized treatment is also required for patients with collapsed femoral head^7^, ^8^. When the necrotic area is mainly confined to the non‐weight‐bearing area, hip preserving procedures can be attempted in patients with a degree of collapse <2 mm. When there is no collapse of the femoral head, the choice of the treatment method should be individualized according to the stage and classification. For femoral head necrosis with small necrotic area located in non‐weight‐bearing areas, medication, core decompression, and extracorporeal shock wave therapy can be used. However, patients with larger necrotic area located in weight‐bearing areas can be treated with PTI, BIG, transtrochanteric rotational osteotomy or vascularized fibula grafting. Our center mainly conducts core decompression, PTI, and BIG surgery through a window at the femoral head neck junction, and the previous studies showed that these two hip preserving procedures can achieve good clinical results^7^, ^8^.

However, total hip arthroplasty is required for some patients due to the progressive collapse of the femoral head after failed hip joint preserving procedure. Conversion to a total hip arthroplasty after failed hip joint preserving procedure is considered a technically challenging procedure in respect of removing the implant, which may result in increased blood loss, bone loss, extended operative time and potential risk of trochanteric fracture. Lee *et al*.^9^ stated that the total surgery duration and blood loss volume in patients who underwent total hip arthroplasty after failed PTI were significantly higher than those in the primary total hip arthroplasty group. Olsen *et al*.^10^ stated that there was no significant difference in postoperative and radiological follow‐up results between total hip arthroplasty after failed PTI and primary total hip arthroplasty group, but the surgery duration and intraoperative blood loss volume were not compared. Rosenwasser *et al*.^11^ reported no technical difficulties while performing total hip arthroplasty after failed BIG using the light bulb procedure for osteonecrosis of the femoral head. Mont *et al*.^12^ conducted the BIG trapdoor procedure using cortical and cancellous bone for treatment of osteonecrosis of the femoral head. In the patients who required conversion into total hip arthroplasty due to the progressive collapse of the femoral head, procedures were conducted without complications related to the previous BIG trapdoor procedure.

Therefore, the main purpose of this retrospective study was to: (i) compare the clinical and radiologic results of total hip arthroplasty after failed PTI, and total hip arthroplasty after failed BIG technique for osteonecrosis of the femoral head with primary total hip arthroplasty at our center; (ii) explore technical difficulties and risks of conversion to a total hip arthroplasty after failed PTI and BIG technique; and (iii) provide perspectives and perceptions for hip joint preserving surgery.

## Materials and Methods

### *Patient Data*

This was a retrospective clinical study. We retrospectively reviewed patients who underwent total hip arthroplasty after failed PTI (group I, 27 patients, 30 hips) between 2010 and 2014 at our institution. Then those patients were 1:1 matched based on age, gender, body mass index (BMI), pre‐operative Harris hip score, and date of the index surgery (±1 year) to patients who underwent total hip arthroplasty after failed BIG (group II, 28 patients, 30 hips) and primary total hip arthroplasty (group III, 30 patients, 30 hips, Table 1).

**Table 1:** Demographics of the three groups

	Group I	Group II	Group III
Variables	(30 cases)	(30 cases)	(30 cases)
Age [years, mean(range)]	41 (21–61)	42 (26–65)	41 (23–63)
Body mass index [kg/m^2^, mean(range)]	24.05 (17.65–31.25)	23.59 (18.62–30.25)	25.45 (17.94–37.52)
Stage before total hip arthroplasty (cases)			
ARCO IIIa	7	4	14
ARCO IIIb/IIIc	15	20	10
ARCO IV	8	6	6
Etiology (cases)			
Idiopathic	3	5	4
Corticosteroid	24	18	21
Alcohol	3	7	5
Follow‐up [months, mean(range)]	64 (52–88)	59 (49–91)	62 (54–85)

Inclusion criteria: (i) patients with osteonecrosis of the femoral head who have undergone PTI or BIG; (ii) progressive collapse of femoral head after PTI or BIG; (iii) conversion to a total hip arthroplasty after failed PTI or BIG; (iv) follow‐up time more than 2 years; (v) complete clinical and radiologic data.

Exclusion criteria: (i) lost to follow‐up within 2 years; (ii) preoperative joint infection cannot be excluded; (iii) incomplete clinical and radiologic data.

The study was approved by the ethics committee of the hospital and was subject to its supervision. Informed consent was obtained from all patients or their family members, and the study conformed to the provisions of the Declaration of Helsinki (as revised in Brazil in 2013).

The clinical evaluation indicators included preoperative and postoperative Harris hip score (HSS) scores, blood loss volume, surgery duration, and complication rate. Radiological follow‐up evaluation indexes included abduction angle and anteversion angle of the acetabular cup, periprosthetic osteolysis, and prosthesis subsidence. The abduction angle and anteversion angle of the acetabular cup was measured using the methods described by Nomura *et al*.^13^ and Widmeretal^14^.

Group I had 30 hips, with the average age of 41 years (range, 21–61 years). The average time from the PTI surgery to conversion to total hip arthroplasty was 31 months (range, 5–66 months). The average follow‐up duration was 64 months (range, 52–88 months). All patients in group I underwent total hip arthroplasty for osteoarthritis of the hip due to the progressive collapse of the femoral head.

Group II had 30 hips, with the average age of 42 years (range, 26–65 years). The average time from the BIG surgery to conversion to total hip arthroplasty was 39 months (range, 3–77 months). The average follow‐up duration was 59 months (range, 49–91 months). All patients in group II underwent total hip arthroplasty for osteoarthritis of the hip due to progressive collapse of the femoral head.

Group III had 30 hips, with the average age of 41 years (range, 23–63 years). Mean follow‐up duration was 62 months (range, 54–85 months). All patients in group III underwent total hip arthroplasty for osteoarthritis of the hip due to progressive collapse of the femoral head.

### *Surgical Procedure*

**Figure 1:**
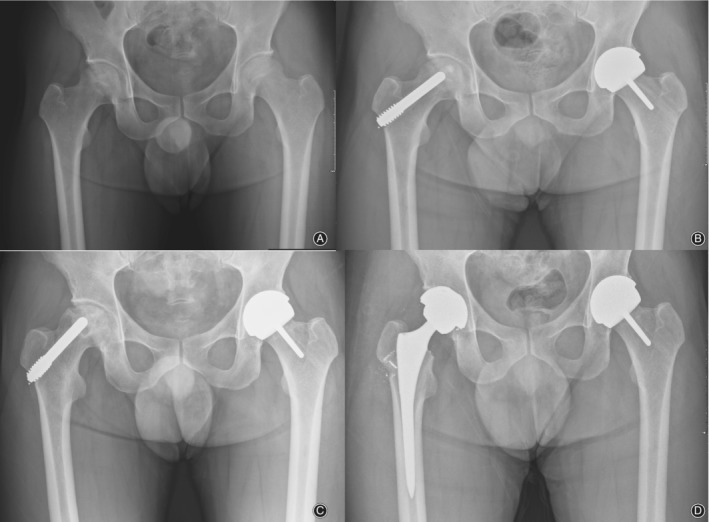
(A) Radiographs of a 46‐year‐old male patient with bilateral avascular necrosis of femoral head. (B) Postoperative radiograph showing that the right femoral head was underwent PTI and the left femoral head was underwent surface replacement. (C) Radiographs taken 12 months post operation show a progressive collapse of the femoral head of the right side. (D) The postoperative radiograph showed remaining metallic particles and bone loss at the lateral femoral cortex of the right side femoral head.

**Figure 2:**
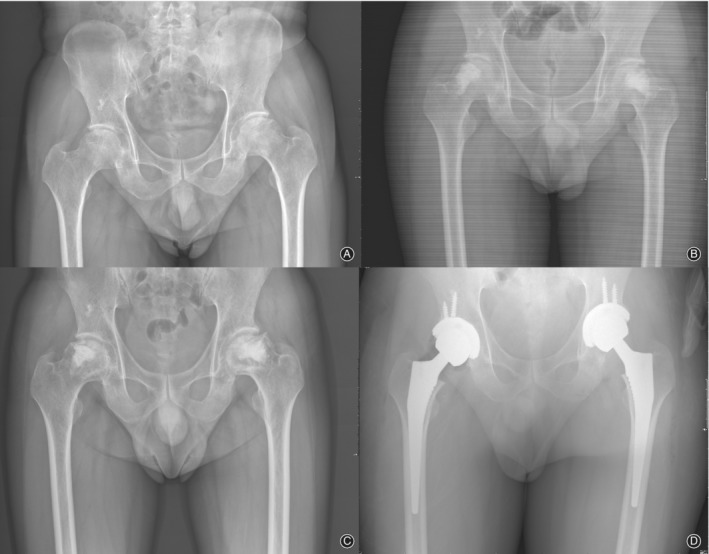
(A) Radiographs of a 36‐year‐old female patient with bilateral avascular necrosis of femoral head. (B) Postoperative radiograph showing that patient was underwent bilateral BIG of the femoral head. (C) Radiographs taken 20 months post operation show a progressive collapse of the bilateral femoral head. (D) Last follow‐up plain radiograph.

**Figure 3:**
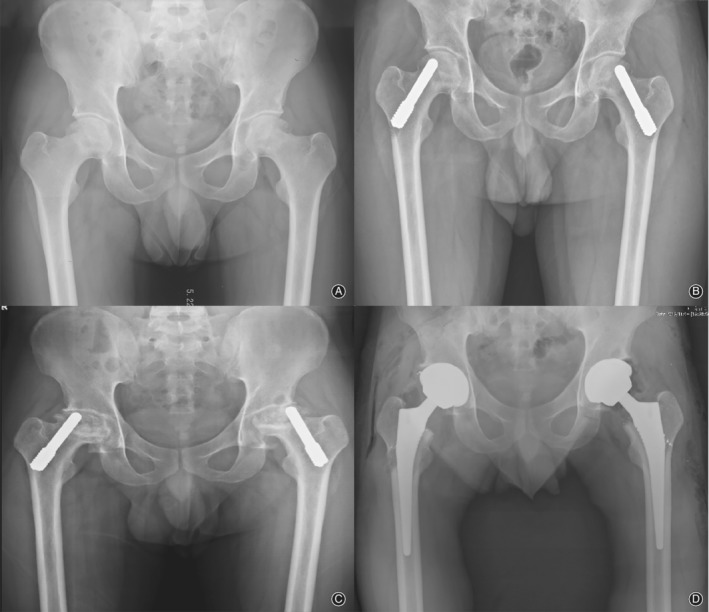
(A) Radiographs of a 29‐year‐old male patient with bilateral avascular necrosis of femoral head. (B) Postoperative radiograph showing that the bilateral femoral head was underwent PTI. (C) Radiographs taken 40 months post operation show a progressive collapse of the femoral head. (D) The postoperative radiograph showed remaining metallic particles and bone loss at the lateral femoral cortex of the right side femoral head.

For patients who underwent total hip arthroplasty after failed tantalum rod implantation, the posterior lateral approach was performed. Femoral head collapse, acetabular cartilage degeneration, and osteophyte hyperplasia were seen during the operation. The femoral neck was cut and the tantalum rod was excised with a pendulum saw. Since the micropores of the tantalum rod have good bone ingrowth, the distal end of the rod had tightly integrated with the femur. The remaining tantalum rod was reamed with a trephine, and was gradually loosened and removed. Then, total hip arthroplasty was performed according to the conventional method. All patients in group I received a bone graft acquired from the femoral head for lateral trochanteric bone defects. For patients who underwent total hip replacement after failed BIG, the posterior lateral approach was performed and then total hip arthroplasty was performed according to the conventional method (Figs 1, 2, 3).

### *Clinical Assessment*

#### *Harris Hip Score (HHS)*

The HHS was used to evaluate postoperative recovery of hip function in adult population. The HHS score system mainly includes four aspects as pain, function, absence of deformity, and range of motion. The score standard had a maximum of 100 points (best possible outcome). A total score < 70 is considered a poor score, 70–80 is fair, 80–90 is good, and 90–100 is excellent.

#### *Inclination Angle (IA)*

The acetabular cup is projected into an ellipse on the frontal X‐ray plates of pelvis. According to the radiological definition of the inclination angle, the angle between the long axis of the ellipse and the bilateral teardrop connection is the radiological definition of the abduction angle.

#### *Acetabular Anteversion Angle (AAT)*

The projection of acetabular cup on anterior and posterior X‐ray film is ellipse. According to the definition of acetabular anteversion angle, the angle between the acetabular axis and coronal plane on an X‐ray is the anteversion angle. Acetabular anteversion angle = arcsin S/L. S is the elliptical short axis and L is the elliptical long axis.

### *Statistical Analysis*

SPSS20.0 was applied to statistical analysis of data. The measurement data were expressed in mean differences ± standard deviation. The counting data were expressed in frequency and percentage. If the measurement data conform to the normal distribution, the three groups are compared by one‐way ANOVA. If the measurement data do not conform to the normal distribution, the nonparametric rank sum test is used. The normality test of the measurement data does not conform to the normality. The nonparametric rank sum test was used in the difference analysis of the three groups. Chi‐square test was used to compare the counting data between groups, the *P* < 0.05 was considered significant.

## Results

### *Clinical Results*

The average follow‐up period for the three groups were 64 (range, 52–88), 59 (range, 49–91), and 62 (range, 54–85) months, respectively (Table 1). The mean preoperative Harris hip score in group I, group II, and group III were 56.50 ± 11.09, 59.07 ± 8.88, and 56.75 ± 6.86 (*P* = 0.313), respectively, which is not significantly different. The postoperative Harris hip score in the three groups were 96.23 ± 1.59, 96.87 ± 2.06, and 96.57 ± 11.09 (*P* = 0.247), respectively, which is also not significantly different.

### *General Results*

The surgery duration of the three groups were 113.17 ± 12.90, 85.00 ± 10.51, and 85.33 ± 9.64 min (*P* < 0.05), respectively, and group I was significantly longer than group II and group III (*P* < 0.05). There was no significant difference between group II and group III. The blood loss volume in the three groups was 570.00 ± 103.06, 405.00 ± 95.01, and 418.33 ± 96.03 mL (*P* < 0.05), respectively, and group I was significantly higher than group II and group III (*P* < 0.05). There was no significant difference between group II and group III (Table 2).

**Table 2:** Comparison of surgical characteristics and clinical outcomesbetween the three groups (mean ± SD)

Characteristics	Group I (30 cases)	Group II (30 cases)	Group III (30 cases)	*Z* value	*P* value
Surgery duration (min)	113.17 ± 12.90	85.00 ± 10.51	85.33 ± 9.64	54.667	0.000
Blood loss (mL)	570.00 ± 103.06	405.00 ± 95.01	418.33 ± 96.03	35.526	0.000
Harris score (preop)	56.50 ± 11.09	59.07 ± 8.88	56.75 ± 6.86	2.322	0.313
Harris score (postop)	96.23 ± 1.59	96.87 ± 2.06	96.57 ± 2.03	2.794	0.247
Acetabular component (°)					
Anteversion	21.23 ± 1.52	21.60 ± 1.79	21.24 ± 2.27	8.747	0.113
Inclination	41.16 ± 3.44	42.63 ± 2.83	41.19 ± 2.60	3.870	0.144

### *Radiographic Results*

The anteversion angle of the acetabular cup of the three groups was 21.23° ± 1.52°, 21.60° ± 1.79°, 21.24° ± 2.27°(*P* = 0.113), respectively, and there was no significant difference between the three groups. The abduction angle of the acetabular cup of the three groups were 41.16° ± 3.44°, 42.63° ± 2.83°, 41.19° ± 2.60° (*P* = 0.144), respectively, and there were no significant differences among the three groups (Table 2).

### *Complications*

One patient in group II developed an intraoperative femoral calcar fracture during broaching and required an additional cable. Posterior dislocation occurred in one patient in group III 2 months after surgery, and they underwent acetabular revision arthroplasty. Both patients achieved good clinical results at the last follow‐up.

## Discussion

Osteonecrosis of the femoral head can be caused by two major types of trauma (fracture of the femoral neck and dislocation of the hip) and non‐trauma. For the rational treatment of osteonecrosis of the femoral head, an individualized plan should be formulated according to the comprehensive consideration of the stages, classification, and age of the patient. Currently, the long‐term effect of total hip arthroplasty in young and middle‐aged patients is not ideal. Therefore, hip preserving procedures should be performed for patients with surgical indications to retain their own joints as much as possible^15^. Hip preserving procedures should comply with the following principles: (i) minimal invasive; (ii) good curative effect, the results are repeatable; and (iii) no increase in difficulties and complications of total hip arthroplasty. The two most commonly used surgical procedures at our institution are PTI and BIG surgery.

### *Total Hip Arthroplasty after Failed PTI*

PTI is a widely used hip preserving procedure. The appropriate surgical indications are the association research circulation osseous (ARCO) I, II and IIIa patients. There are numerous clinical reports about its efficacy^4^, ^5^, ^6^, ^16^, ^17^, ^18^, but few reports of total hip arthroplasty after failed PTI surgery. Lee *et al*.^9^ compared six patients (eight hips) who underwent total hip arthroplasty after failed PTI surgery with 12 patients (16 hips) who underwent primary total hip arthroplasty. The results showed that the total surgery duration and blood loss volume in the PTI group were significantly higher than those in the primary total hip arthroplasty group. The two groups had no significant differences in other clinical and radiological results. Olsen *et al*.^10^ compared 21 patients (21 hips) who underwent total hip arthroplasty after failed PTI surgery with 12 patients (16 hips) who underwent primary total hip arthroplasty. The results showed no significant difference in postoperative and radiological follow‐up results between the two groups, but the surgery duration and intraoperative blood loss volume were not compared. Our results indicated that the surgery duration and intraoperative blood loss volume in the PTI group were significantly higher than those in the BIG group and the primary total hip arthroplasty group, and there was no significant difference between the BIG group and the primary total hip arthroplasty group. The increase in surgery duration and intraoperative blood loss volume were related to the process of removing the tantalum rods. There are various methods for tantalum rod removal. The method used in this study and the majority of previous studies was to cut the femoral neck using an oscillating saw in the standard way, and then a trephine was used to extract the remaining portion in an anterograde fashion. Some surgeons have also used Kirschner wire to drill holes around the tantalum rod, and the tantalum rod was removed after loosening. No matter which method is used to remove the tantalum rod, there are two problems. Firstly, the residue of tantalum debris, followed by the bone defect of the femur. Secondly, the removal of the tantalum rod will inevitably produce lateral femur bone defect, which could lead to periprosthetic fractures and instability of the prosthesis. Lee *et al*.^9^ reported that one out of eight hips developed an intraoperative femoral calcar fracture during broaching and required an additional cable. There was no case of subsidence of the femoral stem during the follow‐up.

### *Total Hip Arthroplasty after Failed BIG*

BIG, through a window at the femoral head neck junction, is also one of the most commonly used hip preserving procedures. Besides mechanical support, it also has decompression effect on the necrotic area, blocking the circulation of ischemia and intraosseous hypertension. This procedure has been used at our hospital for many years, with good clinical effect in the mid‐ and long‐term follow‐up^7^. Rosenwasser *et al*.^11^ reported no technical difficulties while performing total hip arthroplasty after BIG using the lightbulb procedure for osteonecrosis of the femoral head. Mont *et al*.^12^ used trapdoor procedure for the treatment of osteonecrosis of the femoral head, and suggested that this procedure will not affect the total hip arthroplasty after failed BIG surgery. However, none of these studies conducted follow‐up. Our results are similar to their conclusions, the blood loss volume and surgery duration in patients who underwent total hip arthroplasty after failed BIG were significantly less than that in patients who underwent total hip arthroplasty after failed PTI, and there were no significant differences with patients who underwent primary total hip arthroplasty. There were no significant differences between the three groups in the complications and radiological follow‐up results.

### *Metallic Debris Residue*

The large quantities of metallic debris generated by cutting the tantalum rod were commonly observed in the lateral femoral cortex or joint cavity, which might raise concerns about the effect of third‐body particles on wear and an inflammatory foreign body reaction. Lee *et al*.^9^ showed that six of eight hips that underwent total hip arthroplasty after failed PTI surgery had detectable tantalum residues, and one case of squeaking was reported among them. They hypothesized that squeaking was associated with disseminated metallic particles around the prosthesis. Olsen *et al*.^10^ reported that tantalum debris were found in all 21 hips, and their results showed that the existence of tantalum debris does not increase linear wear rate of the highly cross‐linked polyethylene, and the residual amount and distribution area of tantalum debris had no correlation with linear wear rate in short‐term follow‐up. In our study, all 30 hips had detectable residual tantalum debris during postoperative follow‐up. However, there were no complications related to tantalum debris during follow‐up, which needs further clinical follow‐up for confirmation. Fortunately, tantalum is a relatively soft metal and would therefore be less abrasive as a third‐body particle, especially in cases of hard‐hard or those with wear‐resistant highly cross‐linked polyethylene.

### *Conclusion*

This study showed that patients who underwent total hip arthroplasty after failed PTI had higher blood loss volume and surgery duration than patients who underwent total hip arthroplasty after failed BIG and those who underwent primary total hip arthroplasty. Patients who underwent total hip arthroplasty after failed BIG had no significant differences with patients who underwent primary total hip arthroplasty in terms of clinical and radiological follow‐up results. There were no significant differences between the three groups in the HSS scores and radiological follow‐up results.

### *Limitations*

Several limitations in our study should be mentioned. Firstly, data were obtained by retrospective review of medical records. The follow‐up duration was relatively short. Thus, we could not evaluate the long‐term effects of disseminated metallic particles, which could possibly cause osteolysis around the prosthesis. Finally, the sample size is relatively small. However, to our knowledge, this study enrolled the largest number of patients with osteonecrosis of the femoral head under total hip arthroplasty after failed PTI and BIG to date.
